# Supplementary material for: Development of a Simplified Portion Size Selection Task
Source: Foods. 2021 May 18;10(5):1121. doi: 10.3390/foods10051121 (PMC8158092; doi:10.3390/foods10051121)
Supplement: Supplementary file 1 [file foods-10-01121-s001.zip › foods-1195463-supplementary.pdf]

Development of a Simplified Portion Size Selection Task

1.0 Reliability measures of variables.

Hunger: split-half correlations: 1: *r*=.87, 2: *r*=.80, Spearman-Brown: *r*=.91.

Standard PST: split-half correlations: 1: *r*=.83, 2: *r*=.86, Spearman-Brown: *r*=.90.

Slider PST: split-half correlations: 1: *r*=.90, 2: *r*=.88, Spearman-Brown: *r*=.95.

Multiple-Choice PST: split-half correlations: 1: *r*=.90, 2: *r*=.87, Spearman-Brown: *r*=.95.

Liking: split-half correlations: 1: *r*=.71, 2: *r*=.72, Spearman-Brown: *r*=.86.

Filling: split-half correlations: 1: *r*=.71, 2: *r*=.72, Spearman-Brown: *r*=.86.

Familiarity: split-half correlations: 1: *r*=.82, 2: *r*=.85, Spearman-Brown: *r*=.92.

Cognitive Restraint: split-half correlations: 1: *r*=.82, 2: *r*=.57, Spearman-Brown: *r*=.61.

Emotional Eating: split-half correlations: 1: *r*=.90, 2: *r*=.85, Spearman-Brown: *r*=.91.

Uncontrolled Eating: split-half correlations: 1: *r*=.75, 2: *r*=.61, Spearman-Brown: *r*=.77.

Food Insecurity: split-half correlations: 1: *r*=.78, 2: *r*=.82, Spearman-Brown: *r*=.84

2.0 Correlational Analysis

Bonferroni adjusted *p* values were applied to the correlation analysis to counteract issues with multiple comparisons. Overall constructs (e.g. PST, eating behaviors, food insecurity) were used to avoid being too stringent (adjusted *p*=.05/6)=.008). All correlation values are presented in Table S1 and descriptive statistics and correlations for each food presented in Table S2.

Table S1. Descriptive statistics and correlations for portion selection tasks, food characteristics and eating behaviors (*n*=135-150).

	M	*SD*	Range	1.	2.	3.	4.	5.	6.	7.	8.	9.	10.	11
1.SPST	332.23	96.37	113.60-624.51	-										
2.MPST	295.19	101.63	77.60-648.80	.95**	-									
3.SCPST	380.39	114.82	160.80-786.40	.82**	.83**	-								
4.Current Hunger	51.08	24.56	3.75-95.75	.20*	.15	.16*	-							
5.BMI	21.84	3.90	15.30-33.36	.02	.05	.03	-.10	-						
6.Liking	57.42	12.75	18.88-88.16	.54**	.50**	.46**	.13	.03	-					
7.Filling	59.78	10.53	28.96-87.56	.12	.12	.11	.11	.11	.47**	-				
8.Familarity	74.37	14.42	33.36-100.00	.02	.05	.09	-.00	.15	.37**	.39**	-			
9.Uncontrol. Eating	2.38	.53	1.00-3.89	.25**	.19*	.18*	.38**	.10	.28*	.10	.07	-		
10.Cognitive Restraint	2.37	.59	1.00-3.67	-.10	-.12	-.04	-.19*	.17*	.07	.11	.07	.20*	-	
11.Emotional Eating	2.17	.81	1.00-4.00	.06	.01	-.02	.14	.09	.08	.08	-.02	.60**	.23**	-
12.Food Insecurity	1.70	.73	1.00-4.20	.06	.04	.07	.09	-.05	.09	.00	-.13	.25**	.03	.19*

SPST = slider portion selection task, MPST = multiple-choice portion selection task, SCPST = standard computerized portion selection task, Liking = average liking, Filling = average how filling, Familiarity = average familiarity, Uncontrol. Eating = uncontrolled eating. *=*p*<.05, **=*p*<.01 (significant after applying Bonferroni corrections (.05/6=.008).

Table S2. Mean, standard deviations and correlations for individual food items for the three portion selection tasks.

	SCPST	SPST	SCPST/SPST	MPST	SCPST/MPST
	M (*SD*)	*M* (*SD*)	*r*	*M* (*SD*)	*r*
BR	385.87 (185.41)	359.99 (148.08)	.59**	321.60 (154.65)	.60**
CB	338.67 (189.89)	315.92 (132.40)	.61**	256.00 (129.99)	.58**
CC	331.20 (154.37)	296.43 (113.46)	.56**	256.80 (130.38)	.57**
CN	391.73 (193.85)	358.30 (133.27)	.70**	329.60 (144.66)	.73**
CPC	403.20 (201.02)	332.00 (152.99)	.72**	300.00 (177.32)	.70**
CPS	319.73 (205.31)	296.34 (147.44)	.68**	259.20 (165.96)	.73**
CR	517.07 (171.54)	432.27 (118.07)	.58**	432.80 (161.82)	.56**
FF	406.00 (205.23)	356.03 (140.78)	.59**	324.80 (162.51)	.67**
FS	333.87 (214.66)	348.99 (168.98)	.63*	262.40 (172.23)	.68**
GB	351.07 (195.35)	342.08 (162.44)	.72**	310.40 (167.57)	.75**
GBS	566.67 (333.37)	383.79 (200.94)	.66**	372.80 (221.67)	.71**
L	718.53 (234.27)	473.02 (158.85)	.63**	517.60 (172.19)	.66**
MC	536.27 (248.83)	382.06 (142.84)	.71**	365.60 (183.35)	.77**
MM	378.00 (275.23)	304.45 (183.47)	.67**	267.20 (203.01)	.71**
MSD	242.13 (194.34)	266.38 (152.48)	.61**	203.20 (149.54)	.61**
PC	260.00 (123.44)	274.50 (123.85)	.68**	228.80 (233.60)	.69**
PO	214.53 (145.22)	255.06 (151.01)	.71**	202.40 (154.64)	.73**
PP	394.53 (202.45)	380.10 (156.19)	.62**	360.80 (156.73)	.68**
PPTS	254.13 (140.73)	290.05 (126.34)	.62**	233.60 (128.83)	.71**
R	650.67 (246.95)	442.53 (131.16)	.54**	440.80 (156.52)	.55**
RO	435.73 (238.91)	334.35 (167.87)	.63**	311.20 (189.28)	.68**
SBH	216.40 (137.22)	250.69 (109.00)	.41**	188.00 (104.6)	.47**
SR	251.47 (180.49)	251.98 (126.81)	.61**	196.80 (130.94)	.69**
VFR	308.67 (167.18)	312.22 (131.61)	.63**	241.60 (129.48)	.62**
VHKN	292.00	286.21	.47**	232.30	.56**

	(153.61)	(126.43)	(113.30)

CPST = computerised portion selection task, SPST = slider portion selection task, MPST = multiple-choice portion selection task, BR = beef rendang, CB = chicken biriyani, CC = fried carrot cake, CN = chicken nuggets, CPC = chocolate pound cake, CPS = curry puffs, CR = chicken rice, FF = French fries, FS = fruit salad, GB = garlic bread, GBS = green bean soup, L = laksa, MC = mac ‘n’ cheese , MM = M&M’s, MSD = mixed salad with dressing, PC = pandan cake, PO = Pringles original, PP = pepperoni pizza, PPTS = penne pasta with tomato sauce, R = ramen, RO = rojak, SBH = Singapore been hoon, VFR = vegetarian fried rice, VHKN = vegetarian Hong Kong noodles. ** = $p < .01$.

3.0 User Feedback Analysis

Half the participants (50%, *n*=75) preferred the format of the SPST compared to the MPST (36.0%, *n*=54; 14.0% (*n*=21) had no preference). However, in terms of overall ease of use, the MPST (45.1%, *n*=68) was preferred slightly more than the SPST (40.7%, *n*=61; 14.0% (*n*=21) had no preference). The level of preference for ease of distinguishing between portion sizes was similar (SPST: 40.7%, *n*=61; MPST: 44.7%, *n*=67; no preference: 14.7%, *n*=23). Excluding participants that selected no preference, there was no significant difference in the number of participants that chose the SPST or the MSPT; overall preference: χ^2=3.42, *p*=.064, easiest to distinguish portion size: χ^2=.28, *p*=.596, and easiest to use overall: χ^2=.38, *p*=.538. Table S3 presents the feedback characteristics of the SPST and MPST and shows participants rated the MPST as having clearer and more appropriately sized images.

Table S3. Means and standard deviations of the feedback characteristics of the SPST and MPST.

	SPST	MPST	*df*	*t*	*p*	*Hedges g*
Clear images	71.81 (25.14)	78.21 (20.79)	128	-2.60	.010	-.28
Appropriate size images	69.37 (23.40)	74.71 (19.89)	128	-2.28	.011	-.24
Noticeable differences	79.72 (18.83)	77.12 (21.10)	128	1.74	.84	.13
Easy to indicate	74.28 (22.74)	70.08 (21.76)	128	1.51	.133	.19

SPST = slider portion selection task, MPST = multiple choice portion size selection task.

4.0. Open response user feedback.

52 (34.7%) participants provided feedback in the open response question. Some feedback comments referred to the images in general which were applicable for all three versions of the PST. For example, difficulties identifying the depth of bowls used to display soup or noodles, a lack of comparison reference to size of plates used (e.g. participants suggested spoons near the plates for reference) and overall resolution of the images. However, some participants reported the images to be of good quality and commented that viewing them made them hungry and wanted to at the foods they saw. Other generic comments referred to the foods used and suggested a wider range to be captured. In terms of comments that referred to differences in the task, participants tended to prefer the SCPST over the SPST or MPST because they found the SCPST more interactive. Comments also referred to the MPST being difficult to compare the images as they had to scroll over one page and were forced to make a choice. For the slider, comments were made about the images being too small.

5.0. Previous studies within the laboratory group

Study 1: N = 274, females = 177 (64.6%) with average age of 21.55 years old (*SD*=2.05) and average BMI of 21.56 (*SD*=3.39). Average SCPST was 412.92 (*SD*=140.51) with a range of 38.40-847.20 and 1.5% selecting an average portion over 740kcal. No significant difference across sex ($t(270)=1.156$, $p=.249$; males: $M=426.24$, $SD=140.29$; females: $M=405.65$, $SD=140.49$).

Study 2: N = 232, females = 198 (59.6%) with average age of 21.06 years old (*SD*=2.01) and average BMI of 20.94 (*SD*=3.29). Average SCPST was 450.41 (*SD*=122.99) with a range of 143.16-894.51 and 1.2% selecting an average portion over 740kcal. No significant difference

across sex ($t(330)=7.26$, $p<.001$; males: $M=505.81$, $SD=119.59$; females: $M=412.92$, $SD=110.73$).
